# Five-Year Functional Outcomes Among Patients Surviving Aneurysmal Subarachnoid Hemorrhage

**DOI:** 10.1001/jamanetworkopen.2025.1678

**Published:** 2025-03-25

**Authors:** Ho Seok Lee, Min Kyun Sohn, Jongmin Lee, Deog Young Kim, Yong-Il Shin, Gyung-Jae Oh, Yang-Soo Lee, Min Cheol Joo, So Young Lee, Min-Keun Song, Junhee Han, Jeonghoon Ahn, Young-Hoon Lee, Dae Hyun Kim, Young-Taek Kim, Yun-Hee Kim, Won Hyuk Chang

**Affiliations:** 1 Department of Physical and Rehabilitation Medicine, Center for Prevention and Rehabilitation, Heart Vascular Stroke Institute, Samsung Medical Center, Sungkyunkwan University School of Medicine, Seoul, Republic of Korea; 2 Department of Rehabilitation Medicine, College of Medicine, Chungnam National University, Daejeon, Republic of Korea; 3 Department of Rehabilitation Medicine, Konkuk University School of Medicine, Seoul, Republic of Korea; 4 Department and Research Institute of Rehabilitation Medicine, Yonsei University College of Medicine, Seoul, Republic of Korea; 5 Department of Rehabilitation Medicine, Pusan National University School of Medicine, Pusan National University Yangsan Hospital, Yangsan, Republic of Korea; 6 Department of Preventive Medicine, Wonkwang University, School of Medicine, Iksan, Republic of Korea; 7 Department of Rehabilitation Medicine, School of Medicine, Kyungpook National University Hospital, Daegu, Republic of Korea; 8 Department of Rehabilitation Medicine, Wonkwang University School of Medicine, Iksan, Republic of Korea; 9 Department of Rehabilitation Medicine, Jeju National University Hospital, Jeju National University School of Medicine, Jeju City, Republic of Korea; 10 Department of Physical and Rehabilitation Medicine, Chonnam National University Medical School, Gwangju, Republic of Korea; 11 Department of Statistics, Hallym University, Chuncheon, Republic of Korea; 12 Department of Health Convergence, Ewha Womans University, Seoul, Republic of Korea; 13 Department of Preventive Medicine, Chungnam National University Hospital, Daejeon, Republic of Korea; 14 Department of Physical and Rehabilitation Medicine, Sungkyunkwan University School of Medicine, Suwon, Korea; 15 Myongji Choonhye Rehabilitation Hospital, Seoul, Republic of Korea; 16 Department of Health Science and Technology, Samsung Advanced Institute for Health Sciences & Technology, Sungkyunkwan University, Seoul, Republic of Korea; 17 Department of Medical Device Management and Research, Samsung Advanced Institute for Health Sciences & Technology, Sungkyunkwan University, Seoul, Republic of Korea

## Abstract

**Importance:**

Longitudinal changes in functional levels can provide valuable information about disability. However, longitudinal outcomes in aneurysmal subarachnoid hemorrhage (aSAH) have not been well reported, which could provide insight into appropriate management and information for patients experiencing disability.

**Objective:**

To investigate the 5-year prognosis and functional outcomes of patients with aSAH.

**Design, Setting, and Participants:**

This retrospective cohort study used data of patients with aSAH from the Korean Stroke Cohort for Functioning and Rehabilitation study up to 5 years after onset. Data were collected from August 2012 through May 2015 in 9 different hospitals in Korea. Data were analyzed from September 2023 through January 2024.

**Exposure:**

Patients with aSAH surviving at least 7 days after onset.

**Main Outcomes and Measures:**

Assessments were performed serially from 7 days to 5 years after onset. Prognosis, measured by the modified Rankin scale (mRS) in terms of positive outcome (mRS score of 0 or 1), and mortality were analyzed. In addition, sequential functional outcomes were assessed using the Functional Independence Measure (FIM) in survivors of aSAH at 5 years after onset. Multiple imputation method was used to handle missing data. Wilcoxon signed-rank test and paired *t* test were used to analyze differences in functional measurements between each follow-up period. Additionally, a generalized mixed-effects model was used to analyze the longitudinal trajectory of the FIM.

**Results:**

A total of 338 patients with aSAH (mean [SD] age, 56.3 [13.0] years; 207 female [61.2%]) were included. Among survivors of aSAH at 7 days, the 5-year mortality rate was 8.3% (28 participants). The distribution of mRS significantly improved until 4 years and then plateaued, with 180 (53.3%) and 77 (22.8%) patients reporting an mRS score of 0 and 1, respectively. FIM showed a significant improvement up to 4 years (mean [SD] score, 118.9 [18.7]) and then plateaued.

**Conclusions and Relevance:**

In this cohort study, the functional outcomes in patients with aSAH continued to improve up to 4 years after onset, with the majority of participants showing favorable outcomes without significant disability, suggesting that proper long-term assessment is needed and appropriate management should be emphasized to maximize potential outcomes of patients with aSAH.

## Key Points

**Question:**

What is the long-term prognosis in patients with aneurysmal subarachnoid hemorrhage (aSAH)?

**Findings:**

In this cohort study of 338 patients surviving aSAH up to 5 years after onset, functional levels measured by the modified Rankin scale and the Functional Independence Measure showed an improvement up to 4 years and then plateaued.

**Meaning:**

This study assessed longitudinal serial trajectories of functional outcomes in patients with aSAH patients, providing accurate information on recovery patterns and highlighting the importance of appropriate management and rehabilitation to achieve optimal outcomes for both patients and physicians.

## Teaser

This cohort study analyzes long-term functional outcomes among patients who survived aneurysmal subarachnoid hemorrhage (aSAH) in Korea.

## Introduction

Aneurysmal subarachnoid hemorrhage (aSAH) is still a global threatening condition resulting in high morbidity and mortality. A recent study^1^ reported that worldwide incidence of aSAH was 7.9 per 100 000 person-years. Prehospital mortality rates have been reported to be between approximately 22% and 26%, while inpatient mortality rates were reported to be between approximately 19% and 20%.^2^ Furthermore, although reported studies are sparse, approximately 36% to 42% of patients report a poor long-term outcome.^3,4^ Therefore, many previous studies have attempted to predict outcomes of aSAH by reporting on prediction tools.^5,6,7,8^ However, the exact longitudinal trajectories of functional outcomes in aSAH are not well-documented. It is essential to accurately understand the long-term disability and functional outcomes to establish appropriate managing strategies during the recovery phase. Furthermore, because differences in functional levels after initial resuscitation may indicate distinct diseases, understanding variations in functional outcomes is necessary.^9^ Therefore, the aim of this study was to provide analyses of 5-year longitudinal functional outcomes of patients surviving aSAH. A secondary aim was to determine how outcomes vary between different functional levels of patients with aSAH after initial resuscitation.

## Methods

### Data Collection

This cohort study used data from the Korean Stroke Cohort for Functioning and Rehabilitation (KOSCO), a multicenter, prospective cohort of patients with first-time stroke (see the eMethods in Supplement 1 for more information). Data were collected from 9 different hospitals in Korea between August 2012 and May 2015.^10^ All the patients provided written informed consent, and the study protocol was approved by the institutional review board of each participating hospital. This study adhered to the Strengthening the Reporting of Observational Studies in Epidemiology (STROBE) reporting guideline.

This study analyzed data of patients with aSAH from KOSCO up to 5 years after onset. Clinical characteristics including demographic information (age, sex, body mass index [calculated as weight in kilograms divided by height in meters squared], smoking and alcohol history, hypertension, and diabetes) were documented. Comorbidities and prestroke functional level were assessed using the Charlson Weighted Index of Comorbities^11^ and modified Rankin Scale (mRS),^12^ respectively. Glasgow Coma Scale (GCS)^13^ recorded upon the first arrival to the hospital was used to assess severity. Using GCS as a basis, we computed a modified World Federation of Neurosurgical Societies Scale (mWFNS) to assess initial clinical severity.^14^ Whether the patients had concomitant intracerebral hemorrhage or intraventricular hemorrhage was recorded. The specific treatment received by the patients was documented, including endovascular coiling or neurosurgical clipping. Days from onset to treatment were also documented. The following assessments were conducted 7 days after onset: Korean Mini-Mental State Examination (K-MMSE) for cognition^15^ and the Functional Ambulatory Category for ambulation.^16^ The duration of hospitalization, records of whether neurologic symptoms progressed, information regarding medical complications, and receiving inpatient rehabilitation were documented during the first hospitalization. For the inpatient rehabilitation, all neurorehabilitation treatments were applied based on the clinical guidelines in Korea.^17,18^ The complications included thromboembolic disease, pneumonia, ventilator insufficiency, urinary tract infection, and early or late seizure.

Functional outcomes in this study were assessed by 2 measurements. First, the level of disability, as measured by the mRS, was recorded at 7 days and serially from 3 to 60 months after onset. An mRS score of 0 indicated no disability, a score of 1 to 3 indicated mild to moderate disability, a score of 4 to 5 indicated severe disability, and mortality was recorded as an mRS score of 6. Second, functional independence in activities of daily living (ADL) was measured by the Functional Independence Measure (FIM), assessed serially from 3 to 60 months after onset.^19,20^ FIM scores range from 18 to 126, with 18 indicating total dependence and 126 indicating total independence in ADL. All assessments were carried out face to face by licensed occupational and physical therapists and according to the time point specified in the original protocol.^10^ As a quality measure, all assessors were trained through a standardized program that includes both lectures and practical sessions on functional assessments. This program has been conducted every 3 months since the inception of KOSCO in 2012. Additionally, regular audits and data verification processes are in place to ensure data accuracy and consistency.

### Selection of KOSCO Participants With aSAH

**Figure 1.**
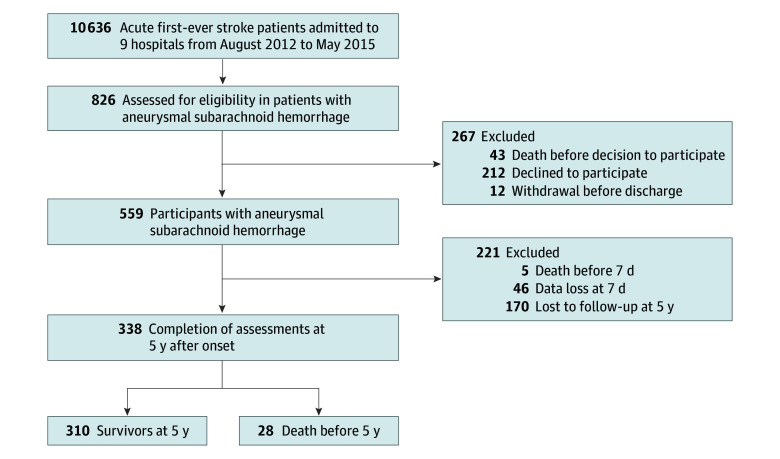
Inclusion Flowchart

Between August 2012 and May 2015, a total of 10 636 patients with first-time stroke were screened. Participants’ eligibility was determined based on their medical records, confirmed by neuroimaging specialists for the presence of aSAH as defined by cerebral angiography. A total of 826 patients with aSAH were screened. The inclusion criteria are as follows: (1) survival at 7 days after onset and (2) completion of assessments at 5 years after onset or death before 5 years after onset. The exclusion criteria are as follows: (1) declined to participate in long-term follow up study, (2) withdrew before discharge, and (3) missing at 7-day or 5-year assessments (Figure 1).

### Statistical Analysis

Demographic and clinical characteristics were reported in terms of frequencies and percentages for categorical variables, while means and SDs were utilized for numerical variables. To classify subgroups, we used an mRS score at 7 days after onset because all patients received specific treatments before 7 days. We defined 2 subgroups as follows: a group with a mild to moderate disability at 7 days as group 1 and a group with severe disability at 7 days as group 2. Group 1 consisted of patients with an mRS score of 0 to 3 at 7 days after onset, while group 2 consisted of patients with an mRS score of 4 or 5.^21,22,23,24^ To compare the characteristics between the subgroups, Fisher exact tests and independent *t* tests were used for categorical and numerical variables, respectively.

For analyses of functional outcomes, Wilcoxon signed-rank tests and paired *t* tests with Bonferroni correction were used to analyze differences in functional measurements between each time point for the mRS and FIM, respectively. Kaplan-Meier survival analysis and Cox proportional hazard regression analysis were performed to evaluate outcomes, as measured by the mRS. To analyze longitudinal trajectories of the FIM, a generalized mixed-effect model was applied (eMethods in Supplement 1). Furthermore, to assess time effects and analyze functional measurements over time among the subgroups, time, subgroups, and interaction between time and subgroups were included as fixed effects. We chose an exponential function for time because recovery patterns are well-known to follow an exponential pattern from previous study.^25^ Age, sex, premorbid hypertension, whether the aneurysm was coiled or clipped, initial mWFNS score, and baseline mRS score and physical impairments were used as covariates in the model for adjustment to account for their potential effects, which were widely known to be associated with functional outcomes of aSAH in previous studies.^6,7,8,26^ Random intercept for patients was modeled and time was also used as a random effect to identify individual changes over time.

Missing data were present during follow-up periods. Multiple imputation was conducted to handle missing data in functional measurements for analyses. Imputation was conducted prior to the aforementioned analyses. We used numerous exposure variables that are known to affect functional levels in previous studies, and therefore we assumed that data were missing at random. The details about the multiple imputation are provided in the eMethods in Supplement 1. Statistical significance was set as a 2-sided *P* < .05 for all the conducted analyses. Statistical analyses were performed using SPSS version 25.0 for Windows (SPSS Inc) from September 2023 to January 2024.

## Results

### Demographics and Clinical Characteristics

Among the 338 included participants, the mean (SD) age was 56.3 (13.0) years and 207 (61.2%) were female. There were 158 participants in group 1 (mild to moderate disability) and 180 participants in group 2 (severe disability). The initial severity, as measured by the mean (SD) GCS and mWFNS scores, were 12.8 (3.5) and 2.2 (1.5), respectively. Compared with group 1, the patients in group 2 were older (mean [SD] age, 59.4 [13.6] years vs 52.9 [11.3] years; *P* < .001) and had worse initial severity (mean [SD] mWFNS score, 2.6 [1.5] vs 1.7 [1.2]; *P* < .001). Group 2 exhibited a higher rate of hypertension than in group 1 (79 participants [43.9%] vs 45 participants [28.5%]; *P* = .005), while other comorbidities showed no differences. All baseline functional and physical impairments were worse in the group 2. Concomitant intraventricular hemorrhage, pneumonia, and ventilator insufficiency during the first hospitalization were more frequently observed in group 2. However, there were no differences between the subgroups in the initial treatment patients received or the time from onset to treatment. Details are provided in Table 1.

**Table 1.** Demographic and Clinical Characteristics of the Patients

Baseline demographic and clinical characteristics	Participants, No. (%)			*P* value^a^
	Total (N = 338)	Group 1: mild to moderate disability (n = 158)	Group 2: severe disability (n = 180)	
Age, mean (SD), y	56.3 (13.0)	52.9 (11.3)	59.4 (13.6)^b^	<.001
Sex				
Male	131 (38.8)	63 (35.0)	68 (43.0)	.15
Female	207 (61.2)	90 (57.0)	117 (65.0)	
Body mass index, mean (SD)^c^	23.5 (3.1)	23.5 (3.0)	23.4 (3.3)	.76
Smoking, current	88 (26.0)	44 (27.8)	44 (24.4)	.53
Alcohol, current	149 (44.1)	77 (48.7)	72 (40.0)	.12
Hypertension	124 (36.7)	45 (28.5)	79 (43.9)^d^	.005
Diabetes	21 (6.2)	6 (3.8)	15 (8.3)	.11
WIC score, mean (SD)	0.6 (0.8)	0.6 (0.8)	0.6 (0.8)	.97
Pre-mRS score, mean (SD)	0.8 (1.6)	0.7 (1.4)	0.9 (1.7)	.34
Initial GCS score, mean (SD)	12.8 (3.5)	13.8 (2.8)	11.9 (3.8)^b^	<.001
Initial mWFNS score, mean (SD)	2.2 (1.5)	1.7 (1.2)	2.6 (1.5)^b^	<.001
High-grade (mWFNS 4 or 5), initial	91 (27.0)	21 (13.3)	70 (39.1)^b^	<.001
Intracerebral hemorrhage	8 (2.4)	4 (2.5)	4 (2.2)	>.99
Intraventricular hemorrhage	33 (9.8)	9 (5.7)	24 (13.3)^d^	.03
Treatment				
Conservative	24 (7.1)	14 (8.9)	10 (5.6)	.29
Coiling	135 (39.9)	65 (41.1)	70 (38.9)	.74
Clipping	159 (47.0)	73 (46.2)	86 (47.8)	.83
Others	20 (5.9)	6 (3.8)	14 (7.8)	.17
Onset to treatment, mean (SD), d				
Conservative	NA	NA	NA	NA
Coiling	0.7 (1.1)	0.8 (1.3)	0.6 (1.0)	.34
Clipping	0.8 (1.2)	0.9 (1.4)	0.8 (1.1)	.50
Other	0.8 (0.8)	1.3 (0.8)	0.7 (0.7)	.11
Functional and physical impairments at 7 d, mean (SD)				
Disability (mRS)	3.4 (1.6)	1.8 (0.9)	4.8 (0.4)^d^	<.001
Cognition (K-MMSE)	16.5 (11.4)	24.4 (6.7)	9.6 (10.1)^d^	<.001
Ambulation (FAC)	1.8 (2.0)	3.5 (1.5)	0.4 (0.9)^d^	<.001
Duration of the first hospitalization, mean (SD) d	34.4 (34.2)	20.2 (13.0)	46.8 (41.5)^d^	<.001
Neurologic progression during the first hospitalization	10 (3.0)	4 (2.5)	6 (3.3)	.76
Inpatient rehabilitation during the first hospitalization	136 (40.2)	42 (26.6)	94 (52.2)^d^	<.001
Complications during the first hospitalization				
Thromboembolic disease	5 (1.5)	2 (1.3)	3 (1.7)	>.99
Pneumonia	21 (6.2)	1 (0.6)	20 (11.1)^d^	<.001
Ventilatory insufficiency	9 (2.7)	1 (0.6)	8 (4.4)^c^	.04
Urinary tract infection	17 (5.0)	8 (5.1)	9(5.0)	>.99
Early seizure	11 (3.3)	3 (1.9)	8 (4.4)	.23
Late seizure	6 (1.8)	1 (0.6)	5 (2.8)	.22
Recurrence during the first hospitalization	6 (1.8)	4 (2.5)	2 (1.1)	.42

Abbreviations: FAC, functional ambulation category; GCS, Glasgow Coma Scale; K-MMSE, Korean version of the Mini-Mental Status Examination; mRS, modified Rankin scale; mWFNS, modified World Federation of Neurological Societies Scale; NA, not applicable; WIC, Charlson Weighted Index of Comorbidities.

^a^ Compared between the subgroups, using Fisher Exact test or independent *t* test.

^b^ *P* < .001 compared with group 1.

^c^ Body mass index was calculated as weight in kilograms divided by height in meters squared.

^d^ *P* < .05 compared with group 1.

### Long-Term Global Outcomes

**Figure 2.**
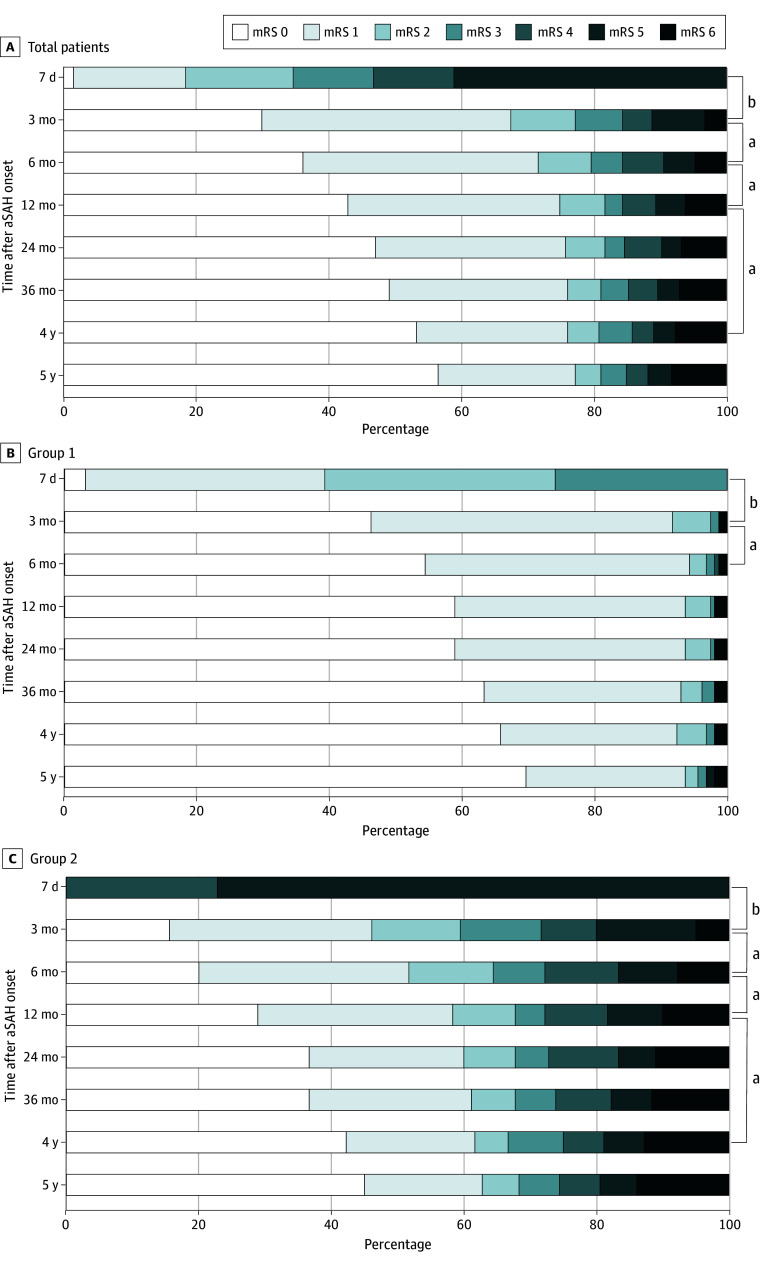
Proportion of Patients Stratified by Modified Rankin Scale (mRS) Over Time An mRS score of 0 indicated no disability, a score of 1 to 3 indicated mild to moderate disability, a score of 4 to 5 indicated severe disability, and a score of 6 indicated mortality. Group 1 consisted of patients with an mRS score of 0 to 3 at 7 days after onset, while group 2 consisted of patients with an mRS score of 4 or 5 at 7 days after onset. *P* values compared between each time point using the Wilcoxon signed-rank test with Bonferroni correction. aSAH indicates aneurysmal subarachnoid hemorrhage. ^a^*P* < .05. ^b^*P* < .001.

In terms of the level of disability, including mortality, the results providing distribution of mRS scores are shown in Figure 2. The mortality among survivors with aSAH at 7 days after onset who completed the 5-year assessments was 3.3% (11 participants) at 3 months, 6.2% (21 participants) at 1 year, and 8.3% (28 participants) at 5 years after onset. The mortality rate at 5 years in group 1 and group 2 was 1.9% (3 participants) and 13.9% (25 participants), respectively. See eFigure 1 and eTable 1 in Supplement 1 for the proportion of mRS values in the original data.

The distribution of mRS scores improved up to 4 years after onset in the overall sample. At 7 days after onset, 5 patients (1.5%) demonstrated no symptoms (mRS = 0) and 57 patients (16.9%) demonstrated no significant disability (mRS = 1). The corresponding proportions were 180 patients (53.3%) with no symptoms and 77 patients (22.8%) with no significant disability at 4 years and 191 patients (56.5%) with no symptoms and 70 patients (20.7%) with no significant disability at 5 years.

In subgroup analysis, group 1 exhibited improvement until 12 months and then plateaued. Meanwhile, in group 2, the distribution of mRS reached a plateau at 4 years. Among patients in group 1 at 12 months, 93 (58.9%) exhibited an mRS score of 0 and 55 (34.8%) exhibited an mRS score of 1. Among patients in group 2 at 12 months, 52 (28.9%) had an mRS score of 0 and 53 (29.4%) had an mRS score of 1. By 4 years after onset, when group 2 reached a plateau, 76 patients (42.2%) and 35 patients (19.4%) demonstrated mRS scores of 0 or 1, respectively.

In survival analysis, groups 1 and 2 showed a significant difference, indicating that group 2 had a substantially higher risk of poor outcomes (HR, 6.39; 95% CI, 4.09-9.99; *P* < .001). In Cox proportional hazard regression analysis, older age (HR, 1.05; 95% CI, 1.03-1.07; *P* < .001) and worse initial severity as measured by mWFNS (HR, 1.21; 95% CI, 1.03-1.43; *P* = .02) were associated with an increased risk of poor outcomes. Additionally, better cognitive function evaluated at 7 days was associated with a reduced risk of poor outcomes (HR, 0.94; 95% CI, 0.90-0.97; *P* < .001). The details are provided in eFigure 2 and eTable 2 in Supplement 1.

### Long-Term Functional Outcomes in Survivors With aSAH at 5 Years After Onset

**Figure 3.**
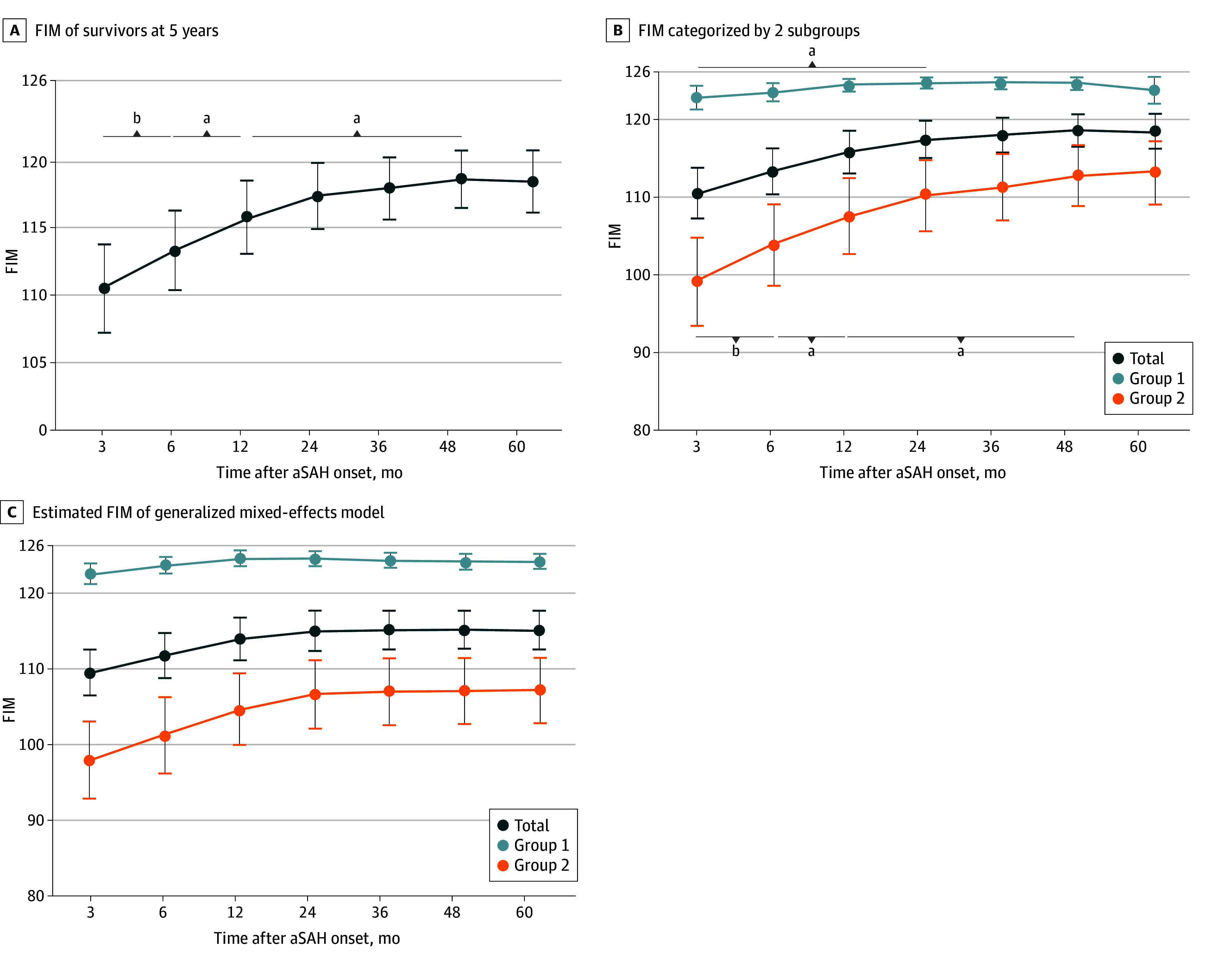
Recovery Patterns of the Functional Independence Measure (FIM) FIM scores range from 18 to 126, with 18 indicating total dependence and 126 indicating total independence in activities of daily living. Group 1 consisted of patients with a modified Rankin scale score of 0 to 3 (mild to moderate disability) at 7 days after onset, while group 2 consisted of patients with an mRS score of 4 or 5 (severe disability) at 7 days after onset. aSAH indicates aneurysmal subarachnoid hemorrhage. ^a^*P* < .05. ^b^*P* < .001, compared between each time point using paired *t* test with Bonferroni correction.

In terms of independence of ADL, the FIM showed improving tendency throughout the follow-up period. At 3 months after onset, the mean (SD) FIM score was 113.5 (26.3). From 3 months onwards, an improvement in the FIM was identified, reaching a mean (SD) FIM score of 118.9 (18.7) by 4 years after onset (Figure 3A). Specifically, the improvement in mean FIM score was 2.4 points from 3 to 6 months (mean [SD] score, 115.9 [23.0]), 1.6 points from 6 to 12 months (mean [SD] score, 117.5 [21.6]), and 1.4 points from 12 months to 4 years after onset.

In subgroup analysis, the FIM in group 1 improved from 3 months (mean [SD] score, 123.0 [9.8]) to 24 months (mean [SD] score, 124.8 [4.1]) after onset (*P* = .02). Meanwhile, the FIM in group 2 improved from 3 months (mean [SD] score, 104.1 [33.4]) to 4 years (mean [SD] score, 113.2 [24.7]) after onset, and reached a plateau (*P* = .02) (Figure 3B). At 5 years after onset, the mean (SD) FIM score of all survivors was 118.5 (20.6). Specifically, the improvement in mean FIM score was 4.2 points from 3 to 6 months (mean [SD] score, 108.3 [29.8]), 2.2 points from 6 to 12 months (mean [SD] score, 110.5 [28.6]), and 2.7 points from 12 months to 4 years after onset. Group 1 and group 2 demonstrated mean (SD) FIM scores of 123.8 (11.2) and 113.1 (25.8), respectively. Details of FIM values are provided in eFigure 3 and eTable 3 in Supplement 1.

In mixed-effect model analysis, significant associations of time (β = 0.16; 95% CI, 0.13 to 0.19), clustered subgroups (β = 0.12; 95% CI, 0.03 to 0.21), and interaction between time and subgroups (β = −0.13; 95% CI, −0.17 to −0.08) with improved FIM scores were identified. Age (β = 0.00; 95% CI, −0.01 to 0.00), initial severity (β = −0.03; 95% CI, −0.05 to −0.01), and cognitive function at 7 days (β = 0.01; 95% CI, 0.00 to 0.01) were also showed significant associations as covariates. The results are shown in Figure 3C and Table 2.

**Table 2.** Generalized Mixed-Effects Model for Longitudinal Trajectory of the Functional Independence Measure

Effect	β (95% CI)	Standard error	*P* value	Variance inflation factor
Intercept	4.70 (4.52 to 4.87)	0.09	<.001	NA
Time	0.16 (0.13 to 0.19)	0.02	<.001	2.00
Subgroup (reference: group 2)	0.12 (0.03 to 0.21)	0.05	.01	3.24
Interaction: time × subgroup (reference: group 2)	−0.13 (−0.17 to −0.08)	0.02	<.001	2.49
Age	0.00 (−0.01 to 0.00)	0.00	.006	1.32
Female sex	0.05 (−0.01 to 0.10)	0.03	.09	1.14
Hypertension	−0.04 (−0.09 to 0.02)	0.03	.16	1.07
Pre-mRS	0.004 (−0.01 to 0.02)	0.01	.63	1.08
Severity (mWFNS), initial	−0.03 (−0.05 to −0.01)	0.01	.001	1.18
Treatment, coiling	−0.01 (−0.09 to 0.07)	0.04	.76	2.63
Treatment, clipping	0.04 (−0.04 to 0.12)	0.04	.37	2.66
Cognitive function (K-MMSE) at 7 d	0.01 (0.00 to 0.01)	0.00	<.001	2.20
Ambulatory function (FAC) at 7 d	0.00 (−0.02 to 0.02)	0.01	.70	2.84

Abbreviations: FAC, functional ambulation category; K-MMSE, Korean version of the Mini-Mental Status Examination; mRS, modified Rankin scale; mWFNS, modified World Federation of Neurosurgery Societies Scale; NA, not applicable.

## Discussion

This cohort study assessed the 5-year longitudinal trajectories of global outcomes and functional levels of patients with aSAH. Both mRS and FIM showed marked improvement up to 4 years and remained plateaued until 5 years after onset. Of the 338 patients with aSAH surviving at 7 days after onset, the 5-year mortality rate was 8.3%. Furthermore, 56.5% of participants displayed no symptoms (mRS = 0) and 20.7% reported no significant disability (mRS = 1) at 5 years.

In previous studies, although none reported serial follow-up of long-term outcomes, the mRS was widely used to assess the functional outcome, including mortality. Previous studies reported that a 1-year and 5-year mortality rate was between approximately 22.1% and 29.0%,^3,4^ and 10.9% and 29.0%,^3,27^ respectively. The mortality rates in our study were 3.3% at 3 months, 6.2% at 1 year, and 8.3% at 5 years after onset. Compared with previous studies,^3,4,27^ the mortality rates in our study were relative low; this was likely attributable to the inclusion criteria of our study, which encompassed patients surviving at 7 days after onset. Indeed, Greebe et al^27^ also reported the mortality rate for patients assessed at 4 months after onset, resulting in a similar 5-year mortality rate to our study. Furthermore, because we targeted all patients admitted to 9 representative hospitals in South Korea and provided long-term serial follow-up data within the same patient group, the result holds substantial importance.

Regarding the positive outcome, the majority of previous studies defined it as an mRS score of 0, 1, or 2. Greebe et al^19^ reported a 5-year positive outcome rate of 60.9%. Other studies reported a 6-month positive outcome rate of 77% and a 1-year positive outcome rate of 58.8%.^4,28^ Roquer et al^3^ demonstrated a positive outcome rate of 57.7% at 3 months, 64.0% at 1 year, and 64.0% at 5 years. Recently, Lider et al^29^ reported the outcomes of patients surviving aSAH at 1 year after onset, with 60% of patients exhibiting a positive functional outcome. Compared with previous studies, the corresponding percentages of positive outcome in this study were as follows: 77.2% at 3 months, 79.6% at 6 months, 81.7% at 12 months, 81.7% at 24 months, 81.1% at 36 months, 80.8% at 4 years, and 81.1% at 5 years after onset. In subgroup analysis, patients in group 1 reached a plateau at 12 months after onset, reporting a 97.5% rate of good outcome. Meanwhile, patients in group 2 reached a plateau 4 years after onset, with 66.6% reporting good outcome. Although no other previous studies report longitudinal change of mRS regarding patients with aSAH, there are some studies involving patients with stroke.^30^ In those studies, patients with stroke reported some decline in function. Therefore, the results of our study may demonstrate different characteristics of aSAH compared with stroke, indicating that function in terms of disability improves in all patients, regardless of their level of function after initial resuscitation. Additionally, patients in group 2 exhibited a higher risk of poor long-term outcomes, with associated factors including age, initial severity, and cognitive function at 7 days.

A sensitive assessment of ADL using the FIM or the Barthel Index is necessary to measure outcomes in patients with aSAH because the mRS may not sufficiently assess ADL sensitivity.^31^ Previous studies reporting outcomes of patients with aSAH using the FIM and the Barthel Index are sparse. Furthermore, these studies only reported the independence of ADL at 1 or 2 time points, making it difficult to predict how functional levels would change over time and provide insights into appropriate management strategies for patients.^32,33,34^ Some other studies reported improvements of the FIM from admission to discharge.^35,36^

In this study, we assessed the longitudinal trajectory of ADL using the FIM up to 5 years after onset, a unique presentation in patients with aSAH. The FIM tended to show significant improvement from 3 months (mean [SD] score, 113.5 [26.3]) to 4 years (mean [SD] score, 118.9 [18.7]) after onset. Meanwhile, there may be some concern that the major improvement in the FIM occurs within the first 12 months. However, the improvement in the FIM from 12 months to 4 years after onset was approximately 26% of the total improvement, similar to the improvement from 6 to 12 months; this suggests that patients with aSAH still have potential to recover from 12 months to 4 years after onset and should not be undertreated. In addition, the exact trajectories of subgroups clustered by functional levels after initial resuscitation were also demonstrated. The FIM of group 1 significantly improved until 24 months, and group 2 improved until 4 years after onset. Consistent with the mRS, independence in ADL also improved for the long term, which differs from the findings of previous studies involving patients with stroke reporting some decline.^37^ Furthermore, in mixed-effect analysis, the associations of time and subgroups were identified. Time (β = 0.16) had a significant association, indicating improvement in the FIM, but with a decreasing rate of improvement over time, eventually reaching a plateau. The interaction between time and subgroups (β = −0.13) was also identified as a significant association, indicating the rate of improvement was rapid in group 2. Among other covariates, age, initial mWFNS, and K-MMSE at 7 days were found to have significant associations. These covariates were consistent with previous studies reporting predictive factors associated with outcomes in patients with aSAH. A notable finding was that baseline cognitive but not ambulatory function was associated with the long-term outcome. This result implies that measurements of cognitive impairments are more essential than measurements of motor impairments in patients with aSAH for predicting the long-term outcome.

### Strengths and Limitations

The strength of this study is that it was a relatively large, multicentered study assessing longitudinal functional outcomes in patients with aSAH. Among the participants analyzed, the missing rate for each time point was less than 12.7% (eTable 4 in Supplement 1) and those missing data were analyzed through multiple imputation.

This study also has some limitations. First, severity based on radiology and clinical severity assessed after resuscitation were lacking. Second, although we found long-term functional improvements, the exact pathophysiology is not revealed. Future studies should focus on understanding the underlying mechanisms and investigating proper rehabilitation strategies to achieve optimal outcomes. Third, functional outcome regarding cognition was not specifically demonstrated. Fourth, although this study was conducted as a multicenter cohort, the participants were exclusively from South Korea. Given that outcomes in patients with aSAH are known to vary based on race or socioeconomic status, the findings of our study may have limited generalizability.^38,39^ Fifth, the number of included patients was relatively modest compared with all eligible patients. However, the aim of this study was to demonstrate the functional outcomes of patients who survived at 7 days after onset. Therefore, we suggest that the results of this study are still meaningful without the patients who died before 7 days or declined to participate. Additionally, we further addressed and compared the baseline characteristics of the excluded and missing patients (eTable 5 and eTable 6 in Supplement 1) in an effort to mitigate selection bias. Although some characteristics differed between the responders and excluded or missing patients during the follow-up periods, the important baseline features such as age, sex, initial severity, functional level at 7 days did not differ.

## Conclusions

In conclusion, this cohort study assessed the long-term trajectory of functional outcomes in patients with aSAH. The functional levels tended to improve up to 4 years after onset, indicating that proper assessment and management should be provided to maximize potential outcomes of patients with aSAH.

## Notes

Supplement 1.
eMethods.

eReferences.
**eFigure 1. **The Proportion of mRS Values in the Original Data **eTable 1. **The Proportion of mRS Values in the Original and Imputed Data **eFigure 2.** Kaplan-Meier Survival Analysis for Predicting Outcomes as Measured by mRS **eTable 2.** Cox Proportional Hazard Regression Analysis for Predicting Outcomes as Measured by mRS **eFigure 3.** The FIM Values in the Original Data **eTable 3.** The Mean Values of FIM in the Original and Imputed Data **eTable 4.** The Proportion of Missing Values for mRS and FIM at Each Time-Point **eTable 5.** Comparison of Demographic and Characteristics Between Responders and Patients Lost to Follow-Up at Each Time-Point **eTable 6. **Comparison of Demographic and Characteristics Between Participants Who Completed Assessments and Excluded Patients Who Were Lost to Follow-Up at 5 Years After Onset

Supplement 2.
Data Sharing Statement
