# Supplementary material for: Five-Year Functional Outcomes Among Patients Surviving Aneurysmal Subarachnoid Hemorrhage
Source: JAMA Netw Open. 2025 Mar 25;8(3):e251678. doi: 10.1001/jamanetworkopen.2025.1678 (PMC11937949; doi:10.1001/jamanetworkopen.2025.1678)
Supplement: Supplement 1. — eMethods. eReferences. eFigure 1. The Proportion of mRS Values in the Original Data eTable 1. The Proportion of mRS Values in the Original and Imputed Data eFigure 2. Kaplan-Meier Survival Analysis for Predicting Outcomes as Measured by mRS eTable 2. Cox Proportional Hazard Regression Analysis for Predicting Outcomes as Measured by mRS eFigure 3. The FIM Values in the Original Data eTable 3. The Mean Values of FIM in the Original and Imputed Data eTable 4. The Proportion of Missing Values for mRS and FIM at Each Time-Point eTable 5. Comparison of Demographic and Characteristics Between Responders and Patients Lost to Follow-Up at Each Time-Point eTable 6. Comparison of Demographic and Characteristics Between Participants Who Completed Assessments and Excluded Patients Who Were Lost to Follow-Up at 5 Years After Onset [file jamanetwopen-e251678-s001.pdf]

Supplemental Online Content

Lee H.S., Sohn M.K., Lee J, et al. Five-Year functional outcomes among patients surviving aneurysmal subarachnoid hemorrhage. *JAMA Netw. Open*. 2025;8(3):e251678.
doi:10.1001/jamanetworkopen.2025.1678

eMethods.

eReferences.

eFigure 1. The Proportion of mRS Values in the Original Data

eTable 1. The Proportion of mRS Values in the Original and Imputed Data

eFigure 2. Kaplan-Meier Survival Analysis for Predicting Outcomes as Measured by mRS

eTable 2. Cox Proportional Hazard Regression Analysis for Predicting Outcomes as Measured by mRS

eFigure 3. The FIM Values in the Original Data

eTable 3. The Mean Values of FIM in the Original and Imputed Data

eTable 4. The Proportion of Missing Values for mRS and FIM at Each Time-Point

eTable 5. Comparison of Demographic and Characteristics Between Responders and Patients Lost to Follow-Up at Each Time-Point

eTable 6. Comparison of Demographic and Characteristics Between Participants Who Completed Assessments and Excluded Patients Who Were Lost to Follow-Up at 5 Years After Onset

This supplemental material has been provided by the authors to give readers additional information about their work.

© 2025 Lee H.S. et al. *JAMA Network Open*.

eMethods.

Original protocol document

This study analyzed data of patients from the Korean Stroke Cohort for Functioning and Rehabilitation (KOSCO). The original protocol document was provided by Chang et al. [1]

Generalized mixed-effect model

To assess the effect of time and analyze functional measurements over time among the subgroups, we plan to use a generalized mixed-effect model. Time and subgroups will be included as fixed effects. Interaction between time and subgroups will also be included as fixed effects. Age, sex, premorbid hypertension, whether the aneurysm was coiled or clipped, initial severity measured by modified World Federation of Neurosurgical Societies scale (mWFNS), and baseline mRS and physical impairments will be used as covariates in the model for adjustment to account for their potential effects. Random intercept for patients was modeled and time was also used as random effect to identify individual changes over time.

We will choose an exponential function for time, as recovery patterns of functional levels are well-known to follow an exponential pattern from previous study. [2]

Distribution of response variable (FIM) will be assumed to follow a Gamma distribution, given that the FIM scores of the participants are continuous and positively skewed. Consequently, the link function will be set to 'log'.

Multiple imputation for missing data

This section describes the multiple imputation used in the analyses. We present key information suggested by Sterne et al. [3]

Missing rate during follow-up periods ranged from 7.4~12.7% (Table S1). Loss to follow-up was defined as missing values of primary outcomes, mRS and FIM scores. The primary reason for missing data was due to patients refusing

© 2025 Lee H.S. et al. *JAMA Network Open*.

the evaluations. We used multiple imputation to estimate values of missing data. We used relatively large number of predictor variables that are known to affect functional levels in previous studies, and therefore we assumed that data were missing at random.

The variable used for predictors and imputed are as follows.

Variables used only as predictors: Age, Sex, Body mass index, Smoking, Alcohol, Hypertension, Diabetes mellitus, Charlson's weighted index of comorbidities (WIC), previous functional level (pre-mRS), initial severity (mWFNS), Death, Neurologic progression, Recurrence, Cognitive function (K-MMSE) at 7 days, Ambulatory function (Functional Ambulatory Category, FAC) at 7 days, and functional level (mRS) at 7 days.

Variables used as predictors and imputed, both: Functional level (mRS) and independence of activities of daily living (FIM) during follow-up periods

The multiple imputation was done using SPSS version 25.0 for Windows (SPSS Inc., Chicago, IL, USA). Since the distribution of data are not normalized, although sample size was relatively large, we performed imputation using a fully conditional specification based on Markov Chain Monte Carlo and predictive mean matching.[4] Five imputations were performed, based on a maximum of 10 iterations.

There was no missing data for variable used only as predictors. The proportions of missing values for mRS and FIM at each time-point during the follow-up, are shown in Table S1 in supplementary appendix. The original values and imputed values or mRS and FIM are provided in Table S2 and S3 in supplementary appendix.

We presented the differences of the baseline characteristics and functional outcomes between the patients with completing assessments and missing in Table S4 in supplementary appendix. We also provided results from analyses restricted to patients completing assessments in Figure S1 and S2 in supplementary appendix.

© 2025 Lee H.S. et al. *JAMA Network Open*.

eReferences

1. Chang WH, Sohn MK, Lee J, et al. Korean Stroke Cohort for functioning and rehabilitation (KOSCO): study rationale and protocol of a multi-centre prospective cohort study. BMC neurology 2015;15(1):1-7.

2. Kwakkel G, Kollen B, Lindeman E. Understanding the pattern of functional recovery after stroke: facts and theories. Restorative neurology and neuroscience 2004;22(3-5):281-99.

3. Sterne JA, White IR, Carlin JB, et al. Multiple imputation for missing data in epidemiological and clinical research: potential and pitfalls. Bmj 2009;338

4. Lee KJ, Carlin JB. Multiple imputation in the presence of non-normal data. Statistics in medicine 2017;36(4):606-17.

eFigure 1. The proportion of mRS values in the original data. Missing values were omitted.

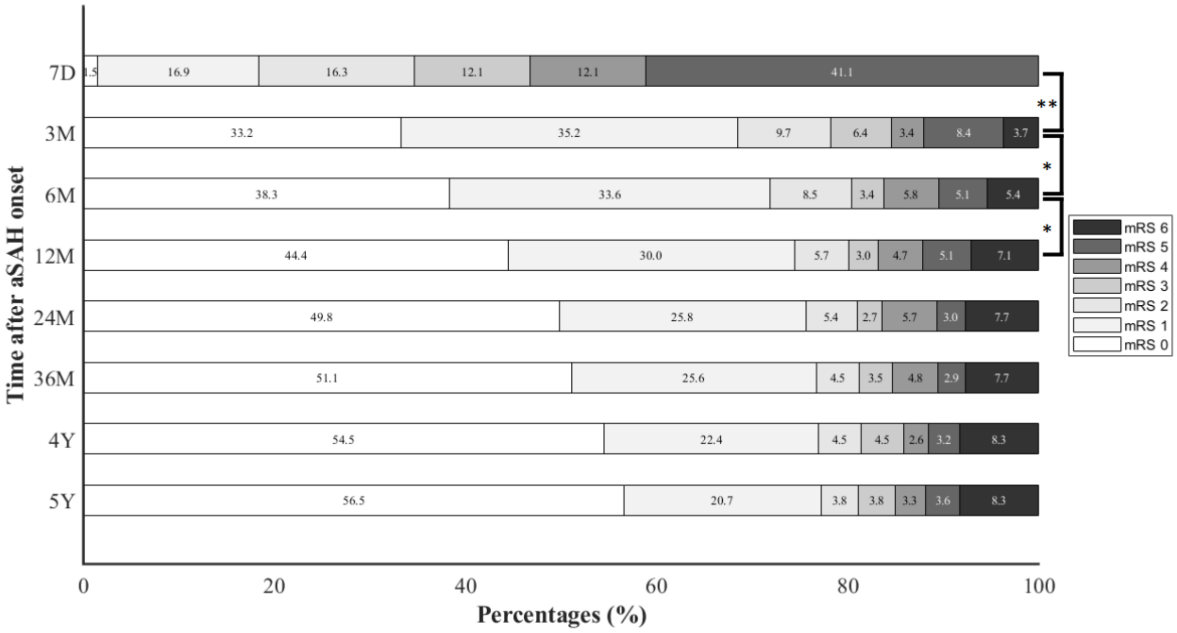

mRS, modified Rankin Scale

*p<0.05, **p<0.001, compared between each time-point using Wilcoxon signed-rank test with Bonferroni correction.

© 2025 Lee H.S. et al. *JAMA Network Open*.

eTable 1. The proportion of mRS values in the original and imputed data. In the original data, missing values were omitted. For the imputed data, the mean, the lowest, and the highest imputed values from five imputations were provided.

		Original data (%)	Imputed data (%)			*p* value[a]
			Mean	Lowest	Highest	
**At 7 days**	mRS 0	1.5	-	-	-	
	mRS 1	16.9	-	-	-	
	mRS 2	16.3	-	-	-	
	mRS 3	12.1	-	-	-	
	mRS 4	12.1	-	-	-	
	mRS 5	41.1	-	-	-	
	mRS 6	0	-	-	-	
**At 3 months**	mRS 0	33.2	29.9	34.9	29.3	0.96
	mRS 1	35.2	37.6	33.4	33.7	
	mRS 2	9.7	9.8	10.7	12.7	
	mRS 3	6.4	7.1	6.5	8.0	
	mRS 4	3.4	4.4	3.6	3.8	
	mRS 5	8.4	8.0	7.7	9.2	
	mRS 6	3.7	3.3	3.3	3.3	
**At 6 months**	mRS 0	38.3	36.1	39.9	33.4	0.97
	mRS 1	33.6	35.5	32.5	34.3	
	mRS 2	8.5	8.0	8.9	10.9	
	mRS 3	3.4	4.7	3.6	3.8	
	mRS 4	5.8	6.2	5.9	6.5	
	mRS 5	5.1	4.7	4.4	6.2	
	mRS 6	5.4	4.7	4.7	4.7	
**At 12 months**	mRS 0	44.4	42.9	48.2	39.6	0.99
	mRS 1	30.0	32.0	27.5	31.7	
	mRS 2	5.7	6.8	5.9	9.5	
	mRS 3	3.0	2.7	3.6	3.6	
	mRS 4	4.7	5.0	4.1	4.7	
	mRS 5	5.1	4.4	4.4	4.7	
	mRS 6	7.1	6.2	6.2	6.2	
**At 24 months**	mRS 0	49.8	47.0	51.8	44.7	0.98
	mRS 1	25.8	28.7	25.1	28.1	
	mRS 2	5.4	5.9	5.0	8.3	
	mRS 3	2.7	3.0	3.3	2.7	
	mRS 4	5.7	5.6	5.3	5.9	
	mRS 5	3.0	3.0	2.7	3.6	
	mRS 6	7.7	6.8	6.8	6.8	
**At 36 months**	mRS 0	51.1	49.1	50.3	47.9	0.99

© 2025 Lee H.S. et al. *JAMA Network Open*.

	mRS 1	25.6	26.9	25.7	26.3	
	mRS 2	4.5	5.0	5.3	6.2	
	mRS 3	3.5	4.1	3.8	4.1	
	mRS 4	4.8	4.4	4.4	5.0	
	mRS 5	2.9	3.3	3.3	3.3	
	mRS 6	7.7	7.1	7.1	7.1	
**At 48 months**	mRS 0	54.5	53.3	53.8	51.5	1.00
	mRS 1	22.4	22.8	22.5	24.0	
	mRS 2	4.5	4.7	5.0	5.0	
	mRS 3	4.5	5.0	5.0	5.0	
	mRS 4	2.6	3.3	3.0	3.3	
	mRS 5	3.2	3.3	3.0	3.6	
	mRS 6	8.3	7.7	7.7	7.7	
**At 60 months**	mRS 0	56.5	-	-	-	
	mRS 1	20.7	-	-	-	
	mRS 2	3.8	-	-	-	
	mRS 3	3.8	-	-	-	
	mRS 4	3.3	-	-	-	
	mRS 5	3.6	-	-	-	
	mRS 6	8.3	-	-	-	

mRS, modified Rankin Scale.

[a] Compared between the original data set and the mean of imputed data set of mRS, using Fisher's Exact test.

© 2025 Lee H.S. et al. *JAMA Network Open*.

eFigure 2. Kaplan-Meier survival analysis for predicting outcomes as measured by mRS.

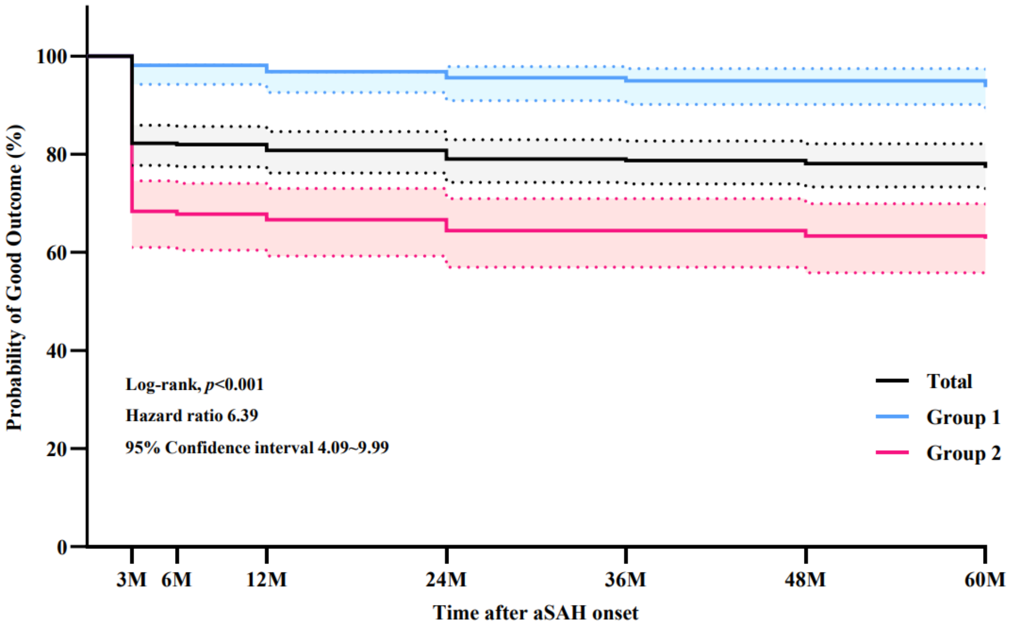

mRS, modified Rankin Scale

© 2025 Lee H.S. et al. *JAMA Network Open*.

eTable 2. Cox proportional hazard regression analysis for predicting outcomes as measured by mRS

Effect	Hazard ratio (95% Confidence interval)	Standard error	*p*-value
Age	1.05 (1.03 - 1.07)	0.01	<0.001
Sex, female	1.38 (0.71 - 2.73)	0.34	0.35
Smoking, yes	1.15 (0.55 - 2.39)	0.38	0.72
Alcohol, yes	0.87 (0.45 - 1.72)	0.34	0.70
Hypertension	1.50 (0.92 - 2.42)	0.25	0.10
Diabetes mellitus	1.53 (0.78 - 2.98)	0.34	0.21
Severity (mWFNS), initial	1.21 (1.03 - 1.43)	0.08	0.02
Treatment, Coiling	0.72 (0.35 - 1.48)	0.37	0.38
Treatment, Clipping	0.77 (0.37 - 1.58)	0.37	0.47
Cognitive function (K-MMSE), at 7-day	0.94 (0.90 - 0.97)	0.02	<0.001
Ambulatory function (FAC), at 7-day	0.80 (0.63 - 1.03)	0.12	0.08

mRS, modified Rankin Scale; mWFNS, modified World Federation of Neurosurgery Societies scale; K-MMSE, Korea version of Mini-Mental Status Examination; FAC, Functional Ambulation Category.

© 2025 Lee H.S. et al. *JAMA Network Open*.

eFigure3. The FIM values in the original data. Missing values were omitted.

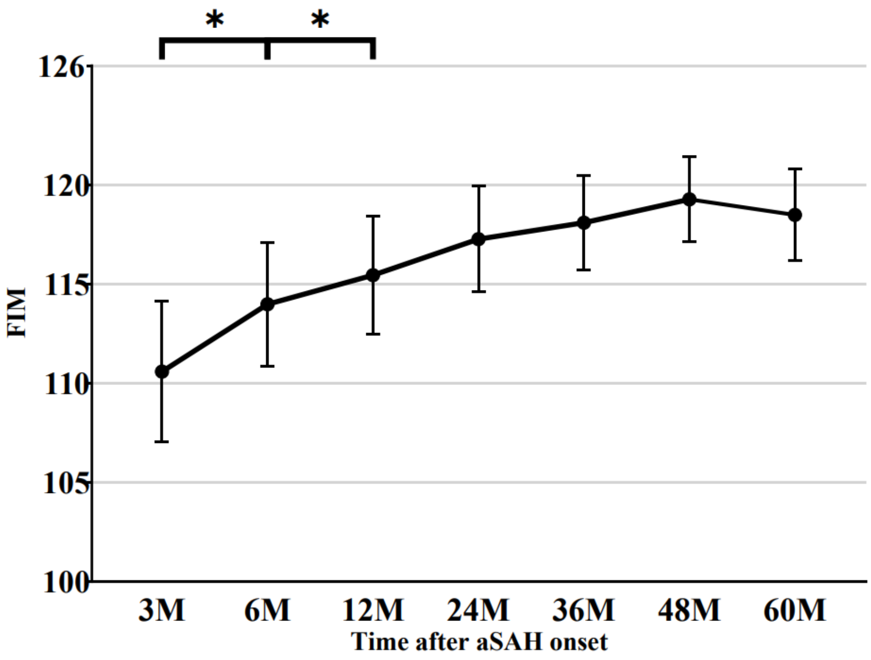

FIM, Functional Independence Measure

**p*<0.05, ***p*<0.001, compared between each time-point using paired t-test with Bonferroni correction.

© 2025 Lee H.S. et al. *JAMA Network Open*.

eTable3. The mean values of FIM in the original and imputed data. In the original data, missing values were omitted. For the imputed data, the mean, the lowest, and the highest imputed values from five imputations were provided.

FIM	Original data (mean[SD])	Imputed data (mean[SD])			*p* value[a]
		Mean	Lowest	Highest	
**Total patients**					
**At 3 months**	113.6±26.4	113.5±26.3	112.6±27.1	114.1±26.0	0.98
**At 6 months**	116.1±22.8	115.9±23.0	115.2±23.6	116.4±22.7	0.91
**At 12 months**	116.9±22.7	117.5±21.6	116.8±22.4	117.9±21.5	0.73
**At 24 months**	118.3±20.7	118.3±20.5	117.9±20.9	118.6±20.2	1.00
**At 36 months**	118.7±19.6	118.5±19.6	118.2±19.8	118.7±19.5	0.90
**At 48 months**	119.6±18.0	118.9±18.7	118.7±19.0	119.1±18.4	0.66
**At 60 months**	118.5±20.6	-	-	-	
**Group 1 – Mild to moderate disability at 7 days after onset**					
**At 3 months**	122.8±10.4	123.0±9.8	122.3±10.2	123.2±9.8	0.90
**At 6 months**	123.9±6.5	123.6±7.4	122.9±8.2	123.8±7.2	0.65
**At 12 months**	124.4±5.1	124.5±4.8	124.1±5.1	124.6±4.8	0.87
**At 24 months**	124.9±4.4	124.8±4.1	124.3±4.6	125.0±4.1	0.88
**At 36 months**	124.8±4.3	124.7±4.5	124.5±4.6	124.8±4.5	0.81
**At 48 months**	124.5±5.1	124.6±4.8	124.5±4.9	124.7±4.8	0.86
**At 60 months**	123.8±11.2	-	-	-	
**Group 2 – Severe disability at 7 days after onset**					
**At 3 months**	104.5±33.4	104.1±33.4	102.9±34.4	105.0±33.1	0.92
**At 6 months**	108.0±29.9	108.3±29.8	107.5±30.5	108.9±29.4	0.93
**At 12 months**	109.3±29.9	110.5±28.6	109.4±29.5	111.1±28.5	0.74
**At 24 months**	112.1±27.2	111.8±27.2	111.4±27.7	112.2±26.8	0.93
**At 36 months**	112.5±26.2	112.3±26.0	111.9±26.2	112.6±25.8	0.97
**At 48 months**	114.7±24.0	113.2±24.7	112.9±25.1	113.6±24.3	0.60
**At 60 months**	113.1±25.8	-	-	-	

FIM, Functional Independence Measure. SD, standard deviation.

[a] Compared between the original data set and the mean of imputed data set of FIM, using independent t-test.

© 2025 Lee H.S. et al. *JAMA Network Open*.

eTable4. The proportion of missing values for mRS and FIM at each time-point

	3 months	6 months	12 months	24 months	36 months	48 months
**mRS**	40 (11.8%)	43 (12.7%)	41 (12.1%)	39 (11.5%)	25 (7.4%)	26 (7.7%)
**FIM**	40 (11.8%)	43 (12.7%)	41 (12.1%)	39 (11.5%)	25 (7.4%)	26 (7.7%)

mRS, modified Rankin Scale; FIM, Functional Independence Measure.

© 2025 Lee H.S. et al. *JAMA Network Open*.

eTable5. Comparison of demographic and characteristics between responders and patients lost to follow-up at each time-point

Baseline demographic and clinical characteristics	At 3 months			At 6 months			At 12 months			At 24 months		
	Respondents (n=298)	Follow-up lost (n=40)	*p*-value[a]	Respondents (n=295)	Follow-up lost (n=43)	*p*-value[a]	Respondents (n=297)	Follow-up lost (n=41)	*p*-value[a]	Respondents (n=299)	Follow-up lost (n=39)	*p*-value[a]
Age	56.5±12.6	55.3±15.7	0.58	56.3±12.9	56.9±13.7	0.77	56.6±12.9	54.4±13.6	0.31	56.5±12.8	55.3±13.9	0.59
Sex, female	177 (59.4%)	30 (75.0%)	0.06	179 (60.7%)	28(65.1%)	0.62	184 (62.0%)	23 (56.1%)	0.50	184 (61.5%)	23 (59.0%)	0.86
Body mass index	23.4±3.1	24.1±3.5	0.19	23.5±3.1	23.2±3.5	0.53	23.4±3.1	24.1±3.4	0.18	23.5±3.2	23.5±3.1	0.95
Smoking, current	80 (26.8%)	8 (20.0%)	0.44	78 (26.4%)	10 (23.3%)	0.71	74 (24.9%)	14 (34.1%)	0.25	78 (26.1%)	10 (25.6%)	1
Alcohol, current	137 (46.0%)	12 (30.0%)	0.06	132 (44.7%)	17 (39.5%)	0.62	131 (44.1%)	18 (43.9%)	1	130 (43.5%)	19 (48.7%)	0.61
Hypertension	110 (36.9%)	14 (35.0%)	0.86	107 (36.3%)	17 (39.5%)	0.74	110 (37.0%)	14 (34.1%)	0.86	112 (37.5%)	12 (30.8%)	0.48
Diabetes mellitus	19 (6.4%)	2 (5.0%)	1	20 (6.8%)	1 (2.3%)	0.49	19 (6.4%)	2 (4.9%)	1	19 (6.4%)	2 (5.1%)	1
WIC	0.6±0.8	0.5±0.7	0.31	0.6±0.8	0.5±0.7	0.35	0.6±0.8	0.4±0.5	0.08	0.6±0.8	0.4±0.5	0.12
pre-mRS	0.9±1.6	0.5±1.4	0.15	0.9±1.6	0.3±0.9	0.02	0.8±1.6	0.7±1.3	0.51	0.8±1.6	0.6±1.3	0.35
GCS score, initial	12.8±3.5	12.8±3.5	0.95	12.7±3.5	13.1±3.2	0.50	12.7±3.6	13.3±2.9	0.32	12.7±3.6	13.4±2.9	0.27
mWFNS	2.2±1.5	2.2±1.5	0.98	2.2±1.5	2.1±1.5	0.70	2.2±1.5	2.1±1.3	0.57	2.2±1.5	2.0±1.3	0.37
High-grade (mWFNS 4 or 5)	80 (26.9%)	11 (27.5%)	1	80 (27.2%)	11 (25.6%)	1	83 (28.0%)	8 (19.5%)	0.35	84 (28.2%)	7 (17.9%)	0.25
Intracerebral hemorrhage	8 (2.7%)	0 (0%)	0.60	7 (2.4%)	1 (2.3%)	1	8 (2.7%)	0 (0%)	0.60	7 (2.3%)	1 (2.6%)	1
Intraventricular hemorrhage	28 (9.4%)	5 (12.5%)	0.57	32 (10.8%)	1 (2.3%)	0.10	32 (10.8%)	1 (2.4%)	0.15	33 (11.0%)	0 (0%)	0.02
Treatment												
Conservative	22 (7.4%)	2 (5.0%)	0.75	23 (7.8%)	1 (2.3%)	0.37	22 (7.4%)	2 (4.9%)	0.75	21 (7.0%)	3 (7.7%)	0.75
Coiling	112 (37.6%)	23 (57.5%)	0.02	119 (40.3%)	16 (37.2%)	0.74	118 (39.7%)	17 (41.5%)	0.87	127 (42.5%)	8 (20.5%)	0.009
Clipping	145 (48.7%)	14 (35.0%)	0.13	134 (45.4%)	25 (58.1%)	0.14	137 (46.1%)	22 (53.7%)	0.41	131 (43.8%)	28 (71.8%)	0.001
Others	19 (6.4%)	1 (2.5%)	0.49	19 (6.4%)	1 (2.3%)	0.49	20 (6.7%)	0 (0%)	0.15	20 (6.7%)	0 (0%)	0.15
Functional and physical impairments at 7-day												
Disability (mRS)	3.4±1.6	3.4±1.7	0.92	3.4±1.6	3.7±1.5	0.16	3.4±1.6	3.3±1.6	0.65	3.4±1.6	3.2±1.4	0.31
Cognition (K-MMSE)	16.3±11.3	17.9±12.1	0.41	16.7±11.4	15.3±11.2	0.47	16.4±11.4	17.8±11.5	0.45	16.2±11.4	19.2±11.2	0.12
Ambulation (FAC)	1.8±2.0	1.9±2.1	0.73	1.8±2.0	1.8±2.0	0.98	1.8±2.0	2.2±2.0	0.24	1.8±2.0	2.2±2.0	0.24
Duration of the 1st hospitalization, days	35.0±35.2	29.6±24.9	0.34	35.6±36.0	26.0±15.8	0.09	36.0±35.7	22.9±15.7	0.02	34.8±33.7	31.1±37.9	0.52
Neurologic progression during the 1st hospitalization	10 (3.4%)	0 (0%)	0.61	9 (3.1%)	1 (2.3%)	1	9 (3.0%)	1 (2.4%)	1	9 (3.0%)	1 (2.6%)	1
Inpatient rehabilitation during the 1st hospitalization	120 (40.3%)	16 (40.0%)	1	123 (41.7%)	13 (30.2%)	0.18	127 (42.8%)	9 (22.0%)	0.01	130 (43.5%)	6 (15.4%)	<0.001
Complications during the 1st hospitalization												
Thromboembolic disease	5 (1.7%)	0 (0%)	1	4 (1.4%)	1 (2.3%)	0.50	5 (1.7%)	0 (0%)	1	5 (1.7%)	0 (0%)	1

© 2025 Lee H.S. et al. *JAMA Network Open*.

Pneumonia	21 (7.0%)	0 (0%)	0.15	21 (7.1%)	0 (0%)	0.09	21 (7.1%)	0 (0%)	0.09	21 (7.0%)	0 (0%)	0.15
Ventilatory insufficiency	8 (2.7%)	1 (2.5%)	1	9 (3.1%)	0 (0%)	0.61	9 (3.0%)	0 (0%)	0.61	9 (3.0%)	0 (0%)	0.61
Urinary tract infection	16 (5.4%)	1 (2.5%)	0.7046	14 (4.7%)	3 (7.0%)	0.46	15 (5.1%)	2 (4.9%)	1	15 (5.0%)	2 (5.1%)	1
Early seizure	10 (3.4%)	1 (2.5%)	1	9 (3.1%)	2 (4.7%)	0.64	11 (3.7%)	0 (0%)	0.37	10 (3.3%)	1 (2.6%)	1
Late seizure	6 (2.0%)	0 (0%)	1	4 (1.4%)	2 (4.7%)	0.17	6 (2.0%)	0 (0%)	1	6 (2.0%)	0 (0%)	1
Recurrence during the 1st hospitalization	5 (1.7%)	1 (2.5%)	0.5331	6 (2.0%)	0 (0%)	1	6 (2.0%)	0 (0%)	1	6 (2.0%)	0 (0%)	1
Functional level at 5-year												
Disability (mRS)	1.2±1.9	1.4±2.1	0.4912	1.2±1.9	1.3±1.9	0.78	1.3±2.0	0.7±1.3	0.06	1.3±2.0	0.8±1.5	0.18
Independence of ADL (FIM)	118.8±20.4	116.2±22.2	0.4901	118.5±20.5	118.3±21.8	0.96	117.8±21.6	122.9±10.5	0.15	118.4±20.9	119.3±18.1	0.80

Continued

© 2025 Lee H.S. et al. *JAMA Network Open*.

Baseline demographic and clinical characteristics	At 36 months			At 48 months		
	Respondents (n=313)	Follow-up lost (n=25)	*p*-value[a]	Respondents (n=312)	Follow-up lost (n=26)	*p*-value[a]
Age	56.6±12.8	53.5±14.6	0.26	56.3±12.9	56.6±13.7	0.91
Sex, female	193 (61.7%)	14 (56.0%)	0.67	190 (60.9%)	17 (65.4%)	0.83
Body mass index	23.4±3.1	23.9±3.6	0.50	23.4±3.1	23.9±3.3	0.47
Smoking, current	81 (25.9%)	7 (28.0%)	0.81	81 (26.0%)	7 (26.9%)	1
Alcohol, current	137 (43.8%)	12 (48.0%)	0.68	139 (44.6%)	10 (38.5%)	0.68
Hypertension	113 (36.1%)	11 (44.0%)	0.52	112 (35.9%)	12 (46.2%)	0.30
Diabetes mellitus	21 (6.7%)	0(0%)	0.38	20 (6.4%)	1 (3.8%)	1
WIC	0.6±0.8	0.4±0.5	0.27	0.6±0.8	0.6±0.6	0.79
pre-mRS	0.8±1.6	0.6±1.3	0.49	0.8±1.5	1.1±1.8	0.29
GCS score, initial	12.8±3.5	12.8±3.2	0.98	12.8±3.5	13.1±3.5	0.65
mWFNS	2.2±1.5	2.4±1.4	0.39	2.2±1.5	2.0±1.6	0.57
High-grade (mWFNS 4 or 5)	84 (26.9%)	7 (28.0%)	1	85 (27.3%)	6 (23.1%)	0.82
Intracerebral hemorrhage	8 (2.6%)	0 (0%)	1	8 (2.6%)	0 (0%)	1
Intraventricular hemorrhage	31 (9.9%)	2 (8.0%)	1	32 (10.3%)	1 (3.8%)	0.49
Treatment						
Conservative	22 (7.0%)	2 (8.0%)	0.70	22 (7.1%)	2 (7.7%)	0.71
Coiling	129 (41.2%)	6 (24.0%)	0.14	128 (41.0%)	7 (26.9%)	0.21
Clipping	143 (45.7%)	16 (64.0%)	0.10	144 (46.2%)	15 (57.7%)	0.31
Others	19 (6.1%)	1 (4.0%)	1	18 (5.8%)	2 (7.7%)	0.66
Functional and physical impairments at 7-day						
Disability (mRS)	3.4±1.6	3.7±1.5	0.37	3.4±1.6	3.1±1.7	0.35
Cognition (K-MMSE)	16.5±11.5	16.6±10.1	0.96	16.5±11.3	17.0±12.5	0.84
Ambulation (FAC)	1.9±2.0	1.4±1.9	0.25	1.8±2.0	1.8±2.1	1
Duration of the 1st hospitalization, days	35.0±35.1	27.0±18.1	0.27	33.1±30.7	49.5±61.3	0.02
Neurologic progression during the 1st hospitalization	9 (2.9%)	1 (4.0%)	0.54	9 (2.9%)	1 (3.8%)	0.56
Inpatient rehabilitation during the 1st hospitalization	129 (41.2%)	7 (28.0%)	0.21	124 (39.7%)	12 (46.2%)	0.54
Complications during the 1st hospitalization						
Thromboembolic disease	5 (1.6%)	0 (0%)	1	5 (1.6%)	0 (0%)	1
Pneumonia	20 (6.4%)	1 (4.0%)	1	20 (6.4%)	1 (3.8%)	1
Ventilatory insufficiency	9(2.9%)	0(0%)	1	8(2.6%)	1(3.8%)	0.52

© 2025 Lee H.S. et al. *JAMA Network Open*.

Urinary tract infection	14(4.5%)	3(12.0%)	0.12	16(5.1%)	1(3.8%)	1
Early seizure	9(2.9%)	2(8.0%)	0.19	11(3.5%)	0(0%)	1
Late seizure	6(1.9%)	0(0%)	1	6(1.9%)	0(0%)	1
Recurrence during the 1st hospitalization	5(1.6%)	1(4.0%)	0.37	5(1.6%)	1(3.8%)	0.38
Functional level at 5-year						
Disability (mRS)	1.2±1.9	1.4±1.8	0.67	1.2±1.9	1.5±1.7	0.48
Independence of ADL (FIM)	118.8±20.2	115.0±25.1	0.39	119.2±19.9	111.0±26.1	0.05

WIC, Charlson's weighted index of comorbidities; mRS, modified Rankin Scale; GCS, Glasgow Coma Scale; mWFNS, modified World Federation of Neurological Societies scale; K-MMSE, Korean version of Mini-Mental Status Examination; FAC, functional ambulation category; FIM, Functional Independence Measure

Values are mean±standard deviations or frequency(percentage).

[a] Compared between the respondents and patients lost to follow-up at each time-point, using Fisher' Exact test of independent t-test.

© 2025 Lee H.S. et al. *JAMA Network Open*.

eTable6. Comparison of demographic and characteristics between participant who completed assessments and excluded patients who were lost to follow-up at 5 years after onset.

Baseline demographic and clinical characteristics	Total (n=508)	Participants (n=338)	Follow-up loss (n=170)	*p*-value[a]
Age	55.9±13.0	56.3±13.0	55.1±13.1	0.32
Sex, female	310 (61.0%)	207 (61.2%)	103 (60.6%)	0.92
Body mass index	23.3±3.2	23.5±3.1	23.0±3.2	0.11
Smoking, current	138 (27.2%)	88 (26.0%)	50 (29.4%)	0.46
Alcohol, current	217 (42.7%)	149 (44.1%)	68 (40.0%)	0.39
Hypertension	183 (36.0%)	124 (36.7%)	59 (34.7%)	0.70
Diabetes mellitus	34 (6.7%)	21 (6.2%)	13 (7.6%)	0.57
WIC	0.6±0.8	0.6±0.8	0.5±0.9	0.73
pre-mRS	0.7±1.4	0.8±1.6	0.4±1.1	0.002
GCS score, initial	12.7±3.5	12.8±3.5	12.5±3.4	0.46
mWFNS, initial	2.3±1.5	2.2±1.5	2.4±1.5	0.11
High-grade (mWFNS 4 or 5), initial	143 (28.2%)	91 (27.0%)	52 (30.6%)	0.40
Intracerebral hemorrhage	13 (2.6%)	8 (2.4%)	5 (2.9%)	0.77
Intraventricular hemorrhage	41 (8.1%)	33 (9.8%)	8 (4.7%)	0.06
Treatment				
Conservative	36 (7.1%)	24 (7.1%)	12 (7.1%)	1
Coiling	186 (36.6%)	135 (39.9%)	51 (30.0%)	0.03
Clipping	259 (51.0%)	159 (47.0%)	100 (58.8%)	0.01
Others	27 (5.3%)	20 (5.9%)	7 (4.1%)	0.53
Onset-to-Treatment, days				
Conservative	NA	NA	NA	NA
Coiling	0.8±1.3	0.7±1.1	0.9±1.5	0.29
Clipping	0.9±1.6	0.8±1.2	1.1±2.0	0.25
Others	1.0±1.0	0.8±0.8	1.4±1.5	0.16
Baseline functional and physical impairments, at 7-day				
Functional level (mRS)	3.4±1.6	3.4±1.6	3.4±1.6	0.99
Cognition (K-MMSE)	16.5±11.5	16.5±11.4	16.6±11.7	0.96
Ambulation (FAC)	1.9±2.0	1.8±2.0	2.0±2.0	0.39
Duration of the 1st hospitalization, days	33.3±32.1	34.4±34.2	31.3±27.3	0.30
Neurologic progression during the 1st hospitalization	21 (4.1%)	10 (3.0%)	11 (6.5%)	0.10
Inpatient rehabilitation during the 1st hospitalization	202 (39.8%)	136 (40.2%)	66 (38.8%)	0.77
Complications during the 1st hospitalization				
Thromboembolic disease	6 (1.2%)	5 (1.5%)	1 (0.6%)	0.67
Pneumonia	36 (7.1%)	21 (6.2%)	15 (8.8%)	0.28

© 2025 Lee H.S. et al. *JAMA Network Open*.

Ventilatory insufficiency	13 (2.6%)	9 (2.7%)	4 (2.4%)	1.00
Urinary tract infection	32 (6.3%)	17 (5.0%)	15 (8.8%)	0.12
Early seizure	21 (4.1%)	11 (3.3%)	10 (5.9%)	0.16
Late seizure	13 (2.6%)	6 (1.8%)	7 (4.1%)	0.14
Recurrence during the 1st hospitalization	8 (1.6%)	6 (1.8%)	2 (1.2%)	0.72

WIC, Charlson's weighted index of comorbidities; mRS, modified Rankin Scale; GCS, Glasgow Coma Scale; WFNS, modified World Federation of Neurological Societies scale; K-MMSE, Korean version of Mini-Mental Status Examination; FAC, functional ambulation category; FIM, Functional Independence Measure

Values are mean±standard deviations or frequency(percentage).

[a] Compared between the participants and patients lost to follow-up at 5 years after onset, using Fisher's Exact test or independent t-test.

© 2025 Lee H.S. et al. *JAMA Network Open*.
