# Supplementary material for: Five-Year Functional Outcomes Among Patients Surviving Aneurysmal Subarachnoid Hemorrhage
Source: JAMA Netw Open. 2025 Mar 25;8(3):e251678. doi: 10.1001/jamanetworkopen.2025.1678 (PMC11937949; doi:10.1001/jamanetworkopen.2025.1678)
Supplement: Supplement 2. — Data Sharing Statement [file jamanetwopen-e251678-s002.pdf]

Data Sharing Statement

Lee. Five-Year Functional Outcomes Among Patients Surviving Aneurysmal Subarachnoid Hemorrhage. *JAMA Netw Open*. Published March 25, 2025. doi:10.1001/jamanetworkopen.2025.1678

Data
Data available: No

Additional Information
Explanation for why data not available: Data will be shared following a reasonable request to e-mail of the corresponding author.
